# Aortic Valve Replacement: Treatment by Sternotomy *versus* Minimally Invasive Approach

**DOI:** 10.5935/1678-9741.20160085

**Published:** 2016

**Authors:** Renata Tosoni Rodrigues Ferreira, Roberto Rocha e Silva, Evaldo Marchi

**Affiliations:** 1 Faculdade de Medicina de Jundiaí (FMJ), Jundiaí, SP, Brazil and Pitangueiras Hospital, Jundiaí, SP, Brazil.; 2 Pitangueiras Hospital, Jundiaí, SP, Brazil; Hospital Paulo Sacramento, Jundiaí, SP, Brazil and Instituto do Coração do Hospital das Clínicas da Faculdade de Medicina da Universidade de São Paulo (InCor-HCFMUSP), São Paulo, SP, Brazil.; 3 Faculdade de Medicina de Jundiaí (FMJ), Jundiaí, SP, Brazil.

**Keywords:** Aortic Valve/Surgery, Aortic Valve Stenosis, Cardiovascular Surgical Procedures, Sternotomy, Cardiopulmonary Bypass, Heart Valve Prosthesis Implantation

## Abstract

**Objective:**

To compare the results of aortic valve replacement with access by sternotomy or minimally invasive approach.

**Methods:**

Retrospective analysis of medical records of 37 patients undergoing aortic valve replacement by sternotomy or minimally invasive approach, with emphasis on the comparison of time of cardiopulmonary bypass and aortic clamping, volume of surgical bleeding, time of mechanical ventilation, need for blood transfusion, incidence of atrial fibrillation, length of stay in intensive care unit, time of hospital discharge, short-term mortality and presence of surgical wound infection.

**Results:**

Sternotomy was used in 22 patients and minimally invasive surgery in 15 patients. The minimally invasive approach had significantly higher time values of cardiopulmonary bypass (114.3±23.9 *versus* 86.7±19.8min.; *P*=0.003), aortic clamping (87.4±19.2 *versus* 61.4±12.9 min.; *P*<0.001) and mechanical ventilation (287.3±138.9 *versus* 153.9±118.6 min.; *P*=0.003). No difference was found in outcomes surgical bleeding volume, need for blood transfusion, incidence of atrial fibrillation, length of stay in intensive care unit and time of hospital discharge. No cases of short-term mortality or surgical wound infection were documented.

**Conclusion:**

The less invasive approach presented with longer times of cardiopulmonary bypass, aortic clamping and mechanical ventilation than sternotomy, however without prejudice to the length of stay in intensive care unit, time of hospital discharge and morbidity.

**Table:** 

**Abbreviations, acronyms & symbols**	
AF	=Atrial fibrillation
AR	=Aortic regurgitation
AS	=Aortic stenosis
AVR	=Aortic valve replacement
B12	=Bleeding in the first 12 hours
B24	=Bleeding in the first 24 hours
BMI	=Body mass index
CPB	=Cardiopulmonary bypass
ICU	=Intensive care unit
MIS	=Minimally invasive surgery

## INTRODUCTION

In Brazil, valve diseases represent a significant number of hospital admissions for cardiovascular diseases, and rheumatic fever is the main cause, responsible for 70% of the cases^[^^1^^]^.

Aortic stenosis (AS) is the aortic valve disease most commonly found and is present in 4.5% of the population over 75 years. The main causes of AS are congenital stenosis, aortic valve calcification, bicuspid or tricuspid stenosis and degenerative rheumatic fever. Rheumatic fever is often associated with mitral valve disease and, despite the reduction in its incidence in developed countries, it is still very common in Brazil and other Latin American countries, particularly in younger patients^[^^1^^]^.

Surgical treatment of the aortic valve is still the only definitive and effective treatment for the relief of left ventricular hypertrophy in patients with severe AS. However, due to surgical risks and early and late complications of prosthetic heart valves, the ideal time for surgery is often considered controversial^[^^2^^]^.

Aortic regurgitation (AR) can be present in aorta dilatation, congenital abnormalities (bicuspid valve), valve calcification, rheumatic disease, infectious endocarditis, hypertension, myxoid degeneration, ascending aortic dissection and Marfan syndrome. Other less common causes include traumatic injuries, ankylosing spondylitis, syphilitic aortitis, rheumatoid arthritis, osteogenesis imperfecta, Ehlers-Danlos syndrome, Reiter's syndrome, subaortic stenosis and ventricular septal defect with aortic cusp prolapse^[^^3^^,^^4^^]^.

Surgical treatment of aortic AR is the choice procedure in symptomatic patients or even in patients with severe ventricular dysfunction, once surgical treatment can lead to an increase in ejection fraction and survival of most patients without progression of the heart failure^[^^1^^,^^5^^]^.

The surgical indications for aortic valve replacement (AVR)are based on evidence levels, and the surgical conventional procedure to AVR is full median sternotomy^[^^6^^]^. Advantages of this method are the complete exposure of the heart and the ascending aorta, with reduction of surgical time, and mortality of this procedure is around 2 to 5%^[^^3^^]^. Clinical outcomes have improved dramatically over the past decades, despite the increased age of patients undergoing this surgery and increased preoperative risks. Recent data from the Society of Thoracic Surgeons database have shown mortality rates of 2.6% and an incidence of stroke of 1.4% in the postoperative period^[^^3^^,^^7^^]^.

Despite numerous advances, several minimally invasive techniques have been developed as alternatives to sternotomy, in order to decrease surgical injury and maintain the same quality and safety of the traditional procedure^[^^3^^]^. According to the American Heart Association, the term "minimally invasive" refers to a small incision in the chest that does not include full sternotomy^[^^3^^]^. Since the first aortic valve replacement through minimally invasive technique, the upper hemisternotomy and right anterior thoracotomy became the predominant accesses for AVR^[^^4^^]^.

In 1996, the initial research of heart valve replacement in canine models demonstrated success in mitral valve replacement through an incision of 2 to 5 cm with cardiopulmonary bypass (CPB). This technique was quickly used in humans in 1997 to replace or repair aortic and mitral valves. Since then, minimally invasive procedures have been practiced with frequency in multiple American institutions^[^^5^^]^.

Although the sternotomy approach is still considered the traditional procedure for aortic valve replacement, in the past 15 years the minimally invasive approach has gained support due to its favorable outcomes^[^^3^^]^. However, further studies evaluating the potential benefits of the minimally invasive approach for AVR are still necessary.

## METHODS

Retrospective analysis of medical records of patients who underwent aortic valve replacement in hospitals of the city of Jundiaí, Brazil, from March 2011 to November 2014. Only adult patients undergoing aortic valve replacement as a single procedure were included. The same team of surgeons performed the procedures.

In the preoperative planning for the minimally invasive surgery (MIS) approach, a chest computed tomography scan was used to assess the anatomy of the intercostal spaces, ascending aorta and aortic valve. Patients eligible for this technique should have: 1- at the level of the main pulmonary artery, at least half of the size of the ascending aorta should be of the right side of the spine, and 2- the distance of the ascending aorta to the sternum should not exceed 10 cm.

Patients with multiple comorbidities and those previously submitted to heart surgery were allocated in the sternotomy group, once according to literature, the minimally invasive approach could increase surgical risks^[^^6^^]^.

MIS was performed under general anesthesia with conventional endotracheal intubation. A horizontal incision of 3-8 cm in the second right intercostal space was performed, followed by muscle dissection, intercostal incision and placement of a small Finochietto retractor. The pericardium was identified, sectioned and pulled by anchor sutures for better exposure of the aorta. Arterial cannulation was performed by direct puncture of the aorta or by dissection and cannulation of the femoral artery. Venous cannulation was performed by direct puncture of the right atrium, with the cannula directed towards the inferior vena cava, or by the femoral access. In the case of cannulation of the aorta, femoral vein cannulation was performed by percutaneous puncture or dissection guided by fluoroscopy. CPB was initiated, and although the smaller access for the MIS approach is technically more difficult, the aortic valve treatment is similar as that performed by sternotomy. At the end of the procedure, after the removal of the cannulas, drainage of the right hemithorax was performed.

The parameters evaluated were:

- Age, gender, body mass index (BMI), history of previous cardiac surgery, type of aortic disease;
- Time of CPB and aortic clamping;
- Bleeding volume in times intraoperative and after 12 and 24 hours;
- Need for blood transfusion; incidence of AF;
- Length of stay in the intensive care unit (ICU) and length of hospital stay;
- Short-term mortality and surgical wound infection indexes.

The software SigmaStat 3.0 (Jandel Scientific, USA) was used for statistical analysis. Student T-test was used to compare the ordinal data between groups sternotomy and MIS, and Chisquare test was used to analyze the nominal data. A *P*<0.05 value was considered significant.

This study was approved by the Research and Ethics Committee of the Jundiaí Medical School (nº. 945.473).

## RESULTS

**Fig. 1:**
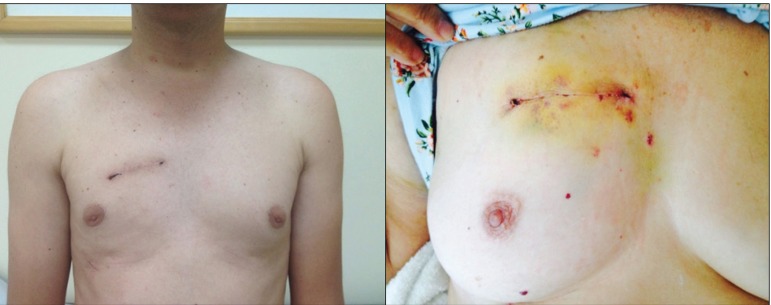
Postoperative aspect of minimally invasive surgery through right mini-thoracotomy for aortic valve replacement.

Out of 37 patients, 22 were approached by sternotomy and 15 by MIS. The appearance of the postoperative incision of the MIS approach is shown in Figure 1.

The age of the sternotomy group was 58.5±16.6 years, and of the MIS group 58.1±17 years, with no statistical difference between groups (*P*=0.816).

Regarding gender, the majority were males (n=26; 70%), of whom 17 underwent sternotomy and 9 the MIS group. Among female patients (n=11; 30%), 5 underwent sternotomy and 6 the MIS. The average BMI was 27.7±4.4 kg/m^2^ for sternotomy and 26.5±2.9 kg/m^2^ for the MIS group (*P*=0.430).

Valve replacement was the first heart surgery for most patients (n=32; 86%). Five (14%) patients with previous surgery for valve replacement were allocated in the sternotomy group.

Double aortic lesion was the most common valve dysfunction found (n=17; 46%), followed by AS (n=11; 30%) and AR (n=9, 24%) (Table 1). Among the 22 patients approached by sternotomy, 13 (59%) patients had double aortic lesion, and among the 15 patients who underwent MIS, 9 (60%) patients had AS.

**Table 1:** Types of aortic valve lesions: patients of sternotomy group (ST) and minimally invasive surgery (MIS).

	**Total**	**ST**	**MIS**
	**N=37**	**N=22 (59%)**	**N=15 (41%)**
Double dysfunction	17 (46%)	13 (59%)	4 (27%)
Aortic stenosis	11 (30%)	2 (9%)	9 (60%)
Aortic regurgitation	9 (24%)	7 (32%)	2 (13%)

The majority of patients received biological aortic prosthesis (n=28; 76%), and only 9 (24%) opted for mechanical prosthesis (2 of them in the MIS group and 7 in the sternotomy group).

Among the 15 patients approached by MIS, in only one (7%) case conversion to sternotomy was necessary due to right ventricular bleeding.

### Times of CPB and Aortic Clamping

**Fig. 2:**
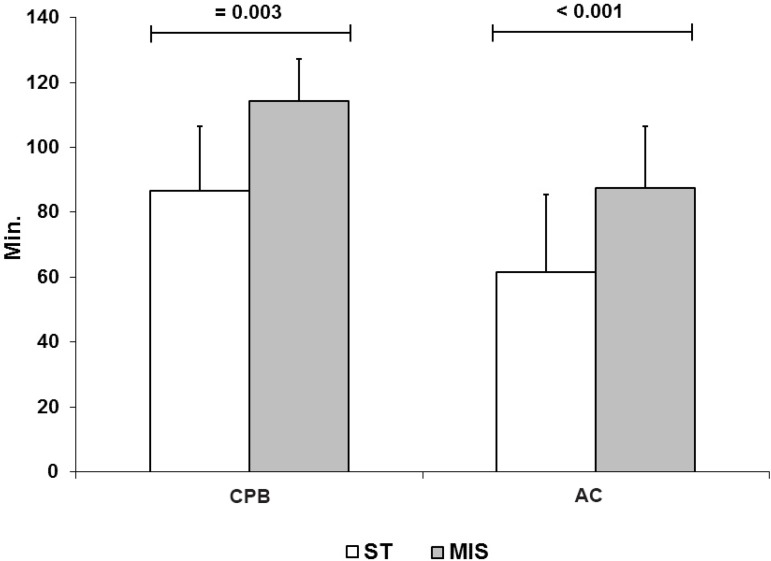
Time (min.) of cardiopulmonary bypass (CPB) and aortic clamping (AC) in the sternotomy group (ST) and minimally invasive surgery (MIS).

The mean CPB time was significantly higher in the MIS approach (114.3±23.9 min.) than for the sternotomy group (86.7±19.8 min.; *P*=0.003). In addition, the mean aortic clamping time was higher for the MIS approach (87.4±19.2 min.) compared to the sternotomy group (61.4±12.9 min.; *P*<0.001) (Figure 2).

### Bleeding Amount

**Fig. 3:**
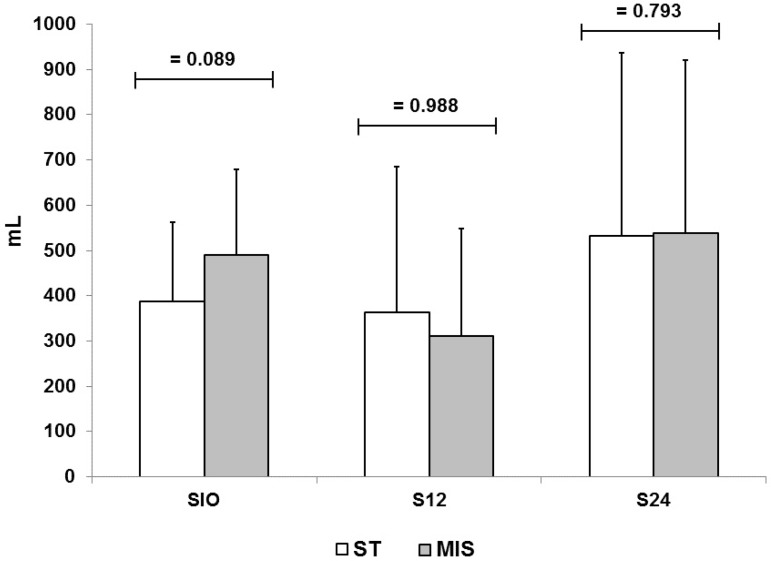
Bleeding (in ml) in the intraoperative period (SIO), 12h (S12) and 24 (S24) of postoperative in ST and MIS groups.

There was no significant difference in the bleeding volume in sternotomy and MIS groups in all time points (Figure 3).

The intraoperative bleeding average was 388.2±174.6 mL for the sternotomy group and 490.7±188.3 mL to the MIS approach (*P*=0.089). Postoperative bleeding in the first 12 hours was 363.3±321.9 mL for the sternotomy group and 310.7±238 mL for the MIS group (*P*=0.988), and in the first 24 hours was 532.2±404 mL for the sternotomy approach and 539.3±381.5 mL to the MIS approach (*P*=0.793).

### Time of Mechanical Ventilation

**Fig. 4:**
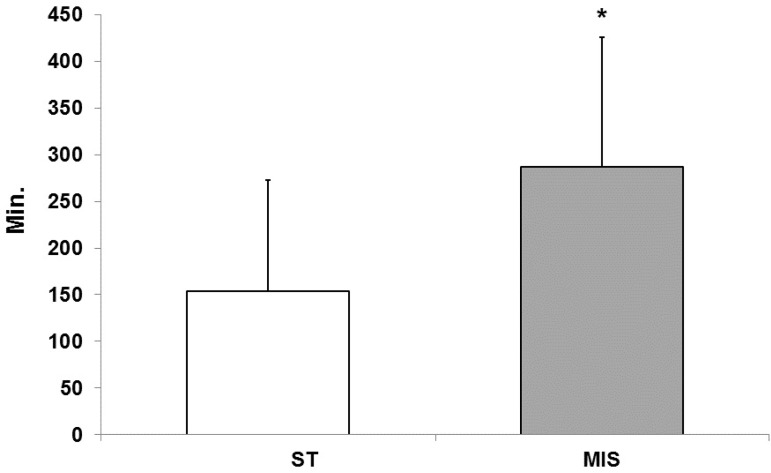
Duration of mechanical ventilation in ST groups.

The mean duration of mechanical ventilation was significantly lower in the sternotomy group (153.9±118.6 min.) compared to the MIS group (287.3±138.9 min.; *P*=0.003) (Figure 4).

### Need for Blood Transfusion

Six (27%) patients of the sternotomy approach group and 2 (13%) of the MIS approach required transfusion, with no statistical difference between both groups (*P*=0.49).

### Incidence of AF

Six (27%) patients of the sternotomy approach and seven (47%) of MIS approach had AF in the postoperative period, with no statistical difference between the groups (*P*=0.92).

### Length of Stay in the ICU and Length of Hospital Stay

The average length of stay in the ICU was 3.9±2.5 days for the sternotomy approach and 3.4±1.2 days for the MIS approach, with no statisticaldifference (*P*=0.975).

The average length of hospital stay was 11±9.0 days for the sternotomy group and 7.1±2.0 days for MIS group, with no statistical difference (*P*=0.454).

### Short-term Mortality and Surgical Wound Infection

Until hospital discharge, no cases of death or wound infection were documented for both approaches.

## DISCUSSION

Aortic valve disease is common in clinical practice and surgical treatment is still the choice procedure for symptomatic patients or patients with important ventricular dysfunction^[^^1^^]^. Median sternotomy is the classic approach for the surgical treatment of aortic valve diseases, but with the technology advancement the MIS approach has been a less invasive alternative to sternotomy^[^^3^^]^. In this sense, further studies are needed to compare the possible favorable outcomes of each procedure in AVR surgery.

The selection of patients that may undergo surgery by sternotomy or the MIS approach depends on a complete preoperative planning, which considers patient comorbidities, the anatomy of the great vessels and the experience of the surgical team^[^^4^^,^^8^^]^. The most common contraindication is the association with other cardiac procedure such as myocardial revascularization. In addition, patients with highly calcified aortic dilatation of the aortic arch, decreased ventricular function, morbidly obese patients, patients with chronic obstructive pulmonary disease, adhesions or pleural thickening in the right hemithorax and previous heart surgery should be considered as relative contraindications^[^^3^^]^.

In patients with multiple comorbidities, the MIS approach can increase the surgical risk due to prolonged CPB and aortic clamping compared to the traditional approach^[^^8^^]^. Isolated comorbidities as cerebrovascular disease, chronic obstructive pulmonary disease, previous radiation therapy in the chest or deformity of the chest wall can also be factors of difficulty to the minithoracotomy approach^[^^6^^]^.

Preoperative planning to MIS approach includes a chest tomography to evaluate the anatomical relationship between the intercostal spaces, ascending aorta and aortic valve^[^^3^^,^^6^^,^^9^^,^^10^^]^.

Adaptation to a new surgical technique, especially with a smaller incision, is undoubtedly accompanied by a surgeon's learning curve. In a study performed at a single center assessing 900 patients undergoing AVR using minimally invasive technique, a decrease of 12% in the CPB time and aortic clamping was observed with the increase of surgeons' experience^[^^5^^]^. According to Plass et al.^[^^11^^]^, since the implementation of the MIS program in the service, it was possible to associate the surgical team learning curve with a lower incidence of complications, duration of CPB and aortic clamping.

Our study followed 37 patients undergoing AVR, 22 undergoing sternotomy approach and 15 to MIS approach (mini-thoracothomy). Our results indicate that both CPB time and aortic clamping time were longer for the MIS approach than for sternotomy, a finding consistent with most of the literature studies^[^^4^^,,^^9^^-^^15^^]^, except in the study by Paredes et al.^[^^16^^]^ that used ministernotomy as a minimally invasive approach, Hiraoka et al.^[^^17^^]^ that used right mini-thoracotomy, and Bakir et al.^[^^18^^]^ that used the inverted J ministernotomy. In other three studies, no significant differences between the two procedures (sternotomy and MIS approach) were found^[^^19^^-^^21^^]^.

The bleeding rate was evaluated in 6 studies, and four of them reported less bleeding volume in the MIS approach^[^^3^^,^^10^^,^^18^^,^^19^^]^. In Hiraoka et al.^[^^22^^]^ and Johnston et al.^[^^5^^]^ studies there was a trend to reduced need for transfusion in the MIS approach, while for Lim et al.^[^^12^^]^ there was no statistically significant difference between the two approaches regarding this parameter.

In the present study, however, a trend for major bleeding was found in the MIS approach, although without statistically significant difference in comparison to sternotomy. It shall be noted that the smaller incision used in minithoracotomy limits the complete exposure of the surgical field, thus increasing the technical difficulty of the procedure, which may have influenced the increased bleeding volume. However, according to the literature, the refinement of the technique according to the surgical team learning curve can contribute to reduce bleeding when MIS is used^[^^5^^,^^11^^]^.

Regarding the time need for mechanical ventilation in the postoperative period, the MIS approach had shorter duration in all studies evaluating this topic^[^^3^^,^^5^^,^^10^^,^^19^^,^^22^^-^^24^^]^, and in three studies there was no statistical difference between the mechanical ventilation in MIS or sternotomy approaches^[^^5^^,^^17^^,^^23^^]^. In our study, the duration of mechanical ventilation was longer in MIS approach than in sternotomy, which contrasts with the literature findings. In this sense, it is possible that because it is an innovative approach in our service, there was some precaution by the ICU team regarding the ventilatory support of patients undergoing MIS.

Moreover, although it was observed a significant difference (*P*=0.003) for mechanical ventilation in the MIS group (287.3±138.9 min. *vs.* 153.9±118.6 min. for sternotomy), this difference was just longer than 2 hours for MIS and did not influence the time for discharge from ICU and hospital length of stay.

The presence of AF has been evaluated in six trials, and the incidence was similar in three of them^[^^10^^,^^21^^,^^23^^]^, while in other three studies there is a decreased incidence of AF for the MIS approach^[^^3^^,^^12^^,^^24^^]^. In our study, there was a higher AF trend in the postoperative period for the MIS approach, but without statistical significance between groups.

Among 14 studies comparing the length of stay in the ICU and length of hospital stay, 10 of them concluded that the MIS approach decreased the length of hospital stay^[^^3^^,^^5^^,^^6^^,^^11^^,^^12^^,^^14^^,^^16^^,^^18^^,^^22^^]^, and among these 7 studies also showed shorter length of stay in the ICU^[^^10^^,^^14^^,^^16^^-^^18^^,^^23^^,^^24^^]^. Four studies did not show significant differences for these parameters between MIS and sternotomy^[^^4^^,^^9^^,^^10^^,^^20^^]^. In accordance with the literature, in our study we also found a tendency for shorter time of stay in the ICU and hospital stay for the MIS approach, although not statistically significant, possibly due to the number of patients evaluated.

Hospital mortality was assessed in 12 studies, and in 10 of them there were no differences between the two approaches^[^^9^^,^^10^^,^^12^^,^^18^^,^^20^^,^^21^ ^23^^,^^25^^,^^26^^]^. In two related studies, there was a reduction in mortality in the MIS approach^[^^4^^,^^16^^]^. In this study, we reported no deaths during the hospital stay.

The wound infection is another topic of great importance evaluated by the literature, in view of its serious consequences, including death by mediastinitis that can occur in cases of infection of the sternotomy incision. According to the studies evaluated, most of them did not show significant differences between the two approaches^[^^3^^,^^8^^,^^16^^]^, and in only one of them there was a lower rate of infection in the MIS approach^[^^21^^]^. In this study, there were no cases of wound infection in any of the approaches.

The main limitations of this study are that it is a retrospective study evaluating a small number of patients. In addition, the number of patients undergoing the MIS approach was limited because it was the initial experience with this surgical approach.

## CONCLUSION

In conclusion, in this study comparing patients undergoing AVR by sternotomy or MIS, the mean time of CPB, aortic clamping and mechanical ventilation were significantly higher in the MIS approach. There was no statistical difference between the two procedures for bleeding in the intraoperative period and after 12 and 24 hours, need for blood transfusion, AF, length of stay in ICU and hospital stay.

**Table:** 

**Authors' roles & responsibilities**	
RTRF	Analysis and/or data interpretation; conception and design study; manuscript redaction or critical review of its content; realization of operations and/or trials; statistical analysis; final manuscript approval
RRS	Conception and design study; manuscript redaction or critical review of its content; final manuscript approval
EM	Conception and design study; manuscript redaction or critical review of its content; statistical analysis; final manuscript approval
